# Experiences of Social Care Among People With Intellectual Disabilities From Minority Ethnic Communities in the UK: A Rapid Review

**DOI:** 10.1111/jar.70283

**Published:** 2026-07-16

**Authors:** Naomi L. Jacobs, Francesca Ribenfors, Christopher Hatton, Lucy Dunstan, Anne‐Marie Glasby

**Affiliations:** ^1^ Manchester Metropolitan University Manchester UK; ^2^ Changing Our Lives Sandwell UK

**Keywords:** cultural competence, intellectual disabilities, intersectionality, minority ethnic communities, rapid review, social care

## Abstract

**Background:**

Adults with intellectual disabilities from minority ethnic communities face intersectional disadvantage in social care. However, there is limited research on social care for these communities, with more studies focused on health care.

**Method:**

This rapid review aimed to map UK research on the social care experiences of adults with intellectual disabilities from minority ethnic communities. A total of 19 studies (2009–2025) were synthesised.

**Results:**

While these communities face similar barriers to social care as majority communities, these are compounded by intersectional disadvantage, including difficulty navigating complex services, experiences of racism and lack of trust, and services that fail to meet cultural and religious needs. Limited support for family carers persists.

**Conclusions:**

The sparse evidence base has limited demographic, topic and methodological diversity. Some evidence argues for relational, person‐centred cultural competence in care. More research is needed on structural and intersectional inequalities, with participation of people with intellectual disabilities from diverse communities.

## Lay Summary

- We looked at research about what social care is like for adults with intellectual disabilities from minority ethnic communities in the UK. We found 19 studies published between 2009 and 2025.
- People with intellectual disabilities from minority ethnic communities face many of the same problems with social care as other people. But they also have extra difficulties. They might deal with racism in social care. Services might not meet their cultural or religious needs. There can be a lack of support for family carers.
- Services need to get to know each person as an individual, rather than making assumptions about what people need based on their background.
- There has not been much research about this subject. We need more studies where researchers ask people with intellectual disabilities from minority ethnic groups about what social care is like for them. People from diverse communities should be involved in research.

## 1 Introduction

There are no reliable statistics on the number of adults with intellectual disabilities from minority ethnic communities who use social care services in the UK (Umpleby et al. 2023). However, research has estimated that by 2030, 25% of adults with intellectual disabilities who are newly receiving social care will be from minority ethnic backgrounds (Emerson et al. 2012). The UK Government's most recent learning disability strategy, *Valuing People Now* (Department of Health 2009), identified people from black and minority ethnic communities as a population facing distinctive health and social care inequalities. Nonetheless, in the 17 years since the strategy's publication, policy reports and research have found continuing inequalities in health and social care for this population (Umpleby et al. 2023; Leeson and Dunstan 2023; Caton et al. 2007; Azmi et al. 1997; Butt and Mirza 1996; O'Hara 2003; Robertson et al. 2019; Devapriam et al. 2008; Burke and Ong 2021; Gregory 2010).

Disabled people from minority ethnic communities experience “double discrimination” (Baxter et al. 1990; Mir et al. 2001), or multiple disadvantage, at the intersection^1^ of disability and ethnic minority status (Butt and Mirza 1996; O'Hara 2003; Mir et al. 2001; Fuentes et al. 2024). Health research demonstrates the substantial impact of these intersecting inequalities, identifying significant barriers to healthcare for people with intellectual disabilities from minority ethnic groups (Umpleby et al. 2023; O'Hara 2003; Robertson et al. 2019), who die significantly younger than their white counterparts (White et al. 2022). The NHS Race and Health Observatory has emphasised the importance of culturally competent care in addressing such health inequalities (Umpleby et al. 2023), noting the importance of training and diversity in workforces and person‐centred care from providers who understand patients' ethnic, cultural and religious backgrounds. In a social care context, it has been argued that culturally competent practice can improve care, promote equality and prevent discrimination, and increase trust in and engagement with services among minority ethnic communities (Lindsay et al. 2014; Theodosopoulos et al. 2024; Care Quality Commission 2025; Laird 2008).

However, research on social care has identified barriers impacting access to and engagement with services for this population and their families, and for other disabled and older users of social care services from minority ethnic communities. Since Ahmad and Atkin's discussion of racism in care services over 20 years ago (Ahmad and Atkin 1996), research has found a lack of cultural competence and discrimination in service settings, with reports of services that fail to meet the cultural, ethnic and religious needs of people from minority ethnic communities, language barriers and poor communication from professionals, a limited awareness of services among those who use social care, and inadequate support for family carers (Leeson and Dunstan 2023; Caton et al. 2007; Azmi et al. 1997; Devapriam et al. 2008; Mir et al. 2001; Laird 2008; Poxton et al. 2012; Hatton et al. 1998; Wilkinson 2009). Research also suggests there are limited policy resources, information and adequate training to help social care providers and commissioners to improve cultural competence (Leeson and Dunstan 2023; O'Hara 2003; Willis et al. 2017), and that there is a need for more evidence‐based research on cultural competence to improve care outcomes (Horevitz et al. 2013; Henderson et al. 2018).

This rapid review was undertaken as part of a larger project undertaking research with people with intellectual disabilities from minority ethnic communities and producing evidence‐based resources to improve cultural competence in supported living and residential care services for adults. The review aims to identify and synthesise existing research on the social care experiences of adults with intellectual disabilities from minority ethnic communities in the UK, since the publication of *Valuing People Now*.

## 2 Methods

### 2.1 Review Approach

Rapid reviews allow for adaptation of standard systematic review methods to a shorter timescale while maintaining methodological rigour (Page et al. 2021; Stevens et al. 2025). Given the relatively sparse prior research on this topic, our review sought to synthesise academic and non‐academic sources, alongside findings from studies where social care may have been considered alongside other settings and services. The SelecTing Approaches for Rapid Reviews (STARR) Decision Tool (Pandor et al. 2019) was used to develop the protocol and search strategy, and PRISMA reporting standards were followed (Page et al. 2021).

### 2.2 Research Question and Objectives

#### 2.2.1 Research Question

The research question was developed by the review team after initial literature searches:

What is known about the social care experiences and needs of adults with intellectual disabilities from minority ethnic communities in the UK, and their families?^2^ What barriers may impede their access to services?

#### 2.2.2 Research Objectives

1. To identify evidence relating to UK social care settings from academic and non‐academic primary research sources.
2. Through systematic synthesis, analysis and reporting, to develop an understanding of the needs for, experiences of and barriers to social care provision for people with intellectual disabilities and family carers from minority ethnic groups.
3. To use the findings to inform an analysis framework for future coproduced research which aims to improve cultural competence in social care services for this population.

### 2.3 Search Strategy

The search strategy was developed with the support of a subject‐specialist academic librarian. Initial searches by the lead reviewer determined the most relevant databases before the search strategy was agreed as a review team. Database selection focused on those most relevant to social care research. Given the limited evidence found in initial searches, multiple academic and unpublished/grey literature databases were used to search for a broad range of evidence.

A systematic search was undertaken in March 2025, using the following academic databases: Applied Social Sciences Indexes and Abstracts (ASSIA), APA PsycInfo, eBook Collection and eBook Open Access Collection, CINAHL, MEDLINE and Sociological Abstracts. Google Scholar was used to support the academic database search, limited to the first five pages of results (Tilley et al. 2023). A search of grey literature was undertaken in April 2025, using the following databases: Carer Research and Knowledge Exchange Network (CAREN), Community Care Inform Adults, the King's Fund Library Database, NHS Knowledge and Library Hub and the Patient Experience Library. Websites of relevant policy and self‐advocacy organisations were also searched.

The search strategy used three concept groups of search terms, relating to (1) intellectual disabilities, (2) social care and (3) minority ethnic communities, using Boolean operators and appropriate truncation, after piloting to refine terms using key databases. Search terms were adapted from literature reviews relevant to social care and intellectual disabilities (Cavanagh et al. 2024; Tilley et al. 2023) and minority ethnic communities (Umpleby et al. 2023). Controlled vocabulary and syntax were modified for each database as appropriate. Geographical terms were used where databases had no geographical limiter functionality. Search terms were adapted to the functionality of each platform; distilled search terms were used for grey literature searches. Comprehensive audit records were maintained.

**FIGURE 1:**
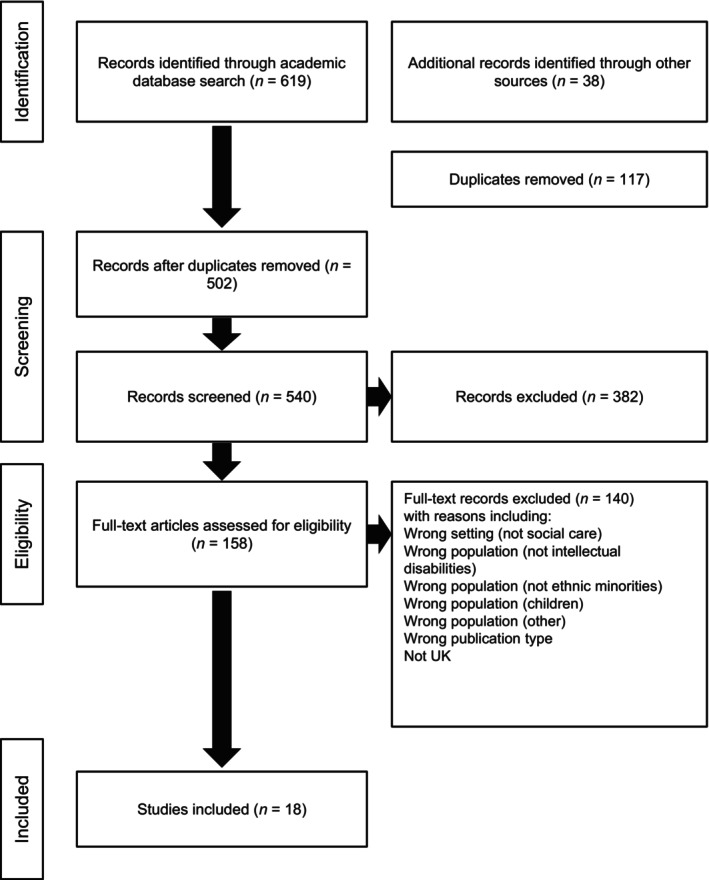
PRISMA flow diagram.

In line with the research project's coproduction methods (Walmsley and Johnson 2003; Corcuff et al. 2025), expert consultation was used to validate grey literature and identify further evidence, following community engagement approaches recommended for rapid reviews (Stevens et al. 2025). Organisations consulted included Learning Disability England, the Race Equality Foundation, Changing Our Lives and academic and policy experts in the field, including members of the project advisory group. Key journals identified during database searches were hand searched; citation tracking was used to identify further relevant evidence from referenced literature in included articles, using CINAHL and MEDLINE to find cited references. One author was contacted by email and provided a cited article that could not be located through the standard search strategy. All evidence identified through these additional methods was screened using the selection process described below and included in the PRISMA flow diagram (Figure 1) under the category ‘records identified from other sources.’

### 2.4 Eligibility Criteria

We reviewed primary evidence which used qualitative, quantitative and mixed methods research designs and case studies. Published and grey literature was included, if it met the criteria below. During the grey literature search, we also looked informally at reports, policy guidance and practice guidance for background and contextual understanding.

#### 2.4.1 Inclusion Criteria

Population: Adults aged 18 years or over with intellectual disabilities from minority ethnic backgrounds and/or their families. Studies with a clearly defined focus on intellectual disabilities and ethnic minority groups were included.

Settings: Social care settings covering a range of service types, including independent and supported living, residential care and day services. Funding models included local authority or NHS‐funded domiciliary care, personal budget arrangements, private ownership and self‐funded social care, and third sector provision. Unpaid family care was only included if it was discussed in relation to statutory social care services. Studies focusing on health care settings were only included if findings on social care were also reported.

Dates of publication: Publications dating from 2009 to 2025 were included to capture evidence reflecting the policy context following the UK Government's most recent comprehensive learning disability strategy *Valuing People Now* (Department of Health 2009), which included policies relating to social care services for people with intellectual disabilities from minority ethnic groups.

#### 2.4.2 Exclusion Criteria

Exclusions: Studies without disaggregated findings for participants with intellectual disabilities and/or participants from minority ethnic communities; studies focusing solely on the experiences of professionals, without involvement of or data relating to people with intellectual disabilities or their families; publications not written in English; incomplete articles; study protocols; secondary reviews and discussion papers (excluded from synthesis but used for citation tracking).

### 2.5 Selection of Evidence

Screening was carried out by the first reviewer using Rayyan systematic review software, with automation features used for deduplication checked manually. Dual independent screening was carried out on 15% of abstracts and full texts by first, second and third reviewers working independently, blinded to each other's decisions (Nussbaumer‐Streit et al. 2023). Inter‐reviewer agreement was 100% at the abstract screening stage, allowing the first reviewer to continue to screen the remaining abstracts. At the full text screening stage, reviewers disagreed about the inclusion of one article (agreement exceeding 90%); resolution was reached through discussion by the review team, following which the remaining full texts were screened by the first reviewer. A researcher with quantitative research expertise validated quantitative article inclusion and analysis (See Figure 1).

### 2.6 Data Extraction and Synthesis

A standardised extraction framework was developed iteratively by the review team, adapting the extraction table used in Umpleby et al. (Umpleby et al. 2023). Extracted data included study focus and service type, research design and methods, participant demographics, emphasising those relevant to the research question, location, age and sample size. Key findings relevant to social care for this population were summarised (See Table 1, ‘Included Sources’).

**TABLE 1:** Included sources.

ID	Authors, year	Focus	Setting	Design	Methods	Key sample characteristics	Location	Age range	Sample size	Key relevant findings
1	Ali et al. (2013)	Barriers to and experiences of health services	Primary health care, hospitals, community intellectual disability services	Qualitative	Semi‐structured interviews; thematic analysis	Adults with mild or moderate intellectual disabilities and their carers interviewed in dyads; participants were White British/White Other (*n* = 9), Asian Indian (*n* = 2) and Asian Pakistani (*n* = 3)	3 London boroughs (Camden and Islington, Newham, Bromley), Somerset and Lincolnshire	23–57	29 (14 parent‐carer dyads, 1 carer)	Found discrimination and barriers persisting despite policy changes. These included a lack of accessible information, complex, opaque systems and poor communication and relationships with professionals. There were also examples of good practice. South Asian participants faced additional challenges including a lack of interpreters and failure to refer to specialist services. Found that misconceptions about extended family support may affect service provision. Recommended that health services offer culturally sensitive care and provide interpreters to reduce language barriers.
2	Bhardwaj et al. (2018)	Social networks and support structures	Day centres, residential care and family homes	Mixed methods	Open‐ended interviews; quantitative analysis of social network data	Adults with intellectual disabilities from White British (*n* = 24) and South Asian (*n* = 23) (Indian, Pakistani, Bangladeshi) backgrounds	London, Dartford and south‐east Kent	19–60	47	Investigated the social networks of two groups of participants with intellectual disabilities. Both groups had similar, but relatively small, average social network sizes. Both networks had personal support made up of paid care and family support, but South Asian participants saw more family daily and more often reported enjoying spending time with family members. It was argued that families may be “gatekeepers” to community participation for this group. More White participants reported enjoying spending time with other service users. More South Asian participants lived at home with family support; more White participants were living independently or in residential care. The majority of staff for both groups were White, but South Asian participants' networks were more ethnically diverse. Found lower self‐sufficiency scores among South Asian participants, which were attributed partly to bilingualism.
3	Bruun et al. (2025)	End‐of‐life care planning	Social care services for people with intellectual disabilities	Qualitative	Focus groups and semi‐structured interviews; framework analysis	People with intellectual disabilities from minoritised ethnic backgrounds, family carers and support staff (White/White British *n* = 14, Black/Black British *n* = 14, Asian/Asian British *n* = 3, mixed background *n* = 3, other/prefer not say *n* = 7; 14 of 25 support staff were White).	UK	20–59+	41 (9 focus groups, 3 individual interviews)	Aimed to address a gap in research on end‐of‐life care planning among people with intellectual disabilities from minoritised ethnic communities. Found that culture and religion had roles in shaping end‐of‐life care preferences. Care planning was seen as culturally dependent. Barriers to planning included taboos around death and disagreement between family members or between families and staff. Emphasised the importance of open conversations and person‐centred, culturally sensitive planning, rather than making assumptions about preferences based on ethnicity. Staff participants highlighted the need for cultural awareness, training and engagement with families and community/religious leaders. Participants often focused on funeral planning, and it was recommended that future research focus on illness‐related planning.
4	Clawson and Fyson (2017)	Forced marriage	UK safeguarding and support services	Mixed methods	Qualitative interviews (*n* = 9); online survey informed by interview themes (*n* = 287); thematic and descriptive statistical analysis	Professionals from services including health, social care, education and police.	UK	N/A	287 survey respondents (71 discussed cases of people with intellectual disabilities); 9 interviews with professionals	Found that forced marriage of people with learning disabilities differs from general patterns: equal risk for men and women and an older age profile. Most marriages took place overseas. Cases were reported across ethnic groups but predominantly in South Asian communities, although this may reflect some reporter bias. Professionals linked forced marriage to families' desire to provide culturally appropriate care and financial security for relatives. Some professionals suggested different cultural concepts of disability and consent were contributing factors. The study highlights the need for improved professional awareness of forced marriage. It notes the importance of avoiding cultural relativism and stereotyping in safeguarding responses.
5	Clawson et al. (2020)	Forced marriage	UK Forced Marriage Unit (FMU) data; cases reported by professionals including from social care and health services	Mixed methods	Secondary analysis of FMU demographic data from case notes; descriptive statistics; limited qualitative insights from additional case notes	FMU data (2009–2015)	UK; highest number of cases from London, West Midlands, south‐east and north‐west England	12–85 (ages represented in cases)	554 cases involving people with intellectual disabilities reported to FMU between 2009 and 2015.	Analysis of UK FMU data comparing demographics of forced marriage involving people with and without intellectual disabilities, with some qualitative analysis of case notes. People with intellectual disabilities have a five times greater risk of forced marriage than the general population, and there are different age and gender patterns, including an equal risk for men and women and different trends in age profiles—up to a quarter of cases are among those aged 31 or older. Most cases were linked to South Asian communities, especially Pakistani communities (45.8% of cases across 3 years). Forced marriage may be linked to a desire for families to secure long‐term care. The majority of reports came from professionals, especially social services (35.4%). Forced marriage of people with intellectual disabilities was found to be a safeguarding issue, but statutory safeguarding guidance does not discuss forced marriage for this population.
6	Cooper‐Moss et al. (2025)	Barriers to health services	Health care including GP practices, hospitals and community services	Qualitative	Experience‐Based Co‐Design (EBCD) workshops; framework analysis	Self‐advocates: 9 Black (African, Caribbean, British), 1 Chinese, 1 mixed ethnic background, 2 undisclosed; 7 male, 4 female. Carers: all 5 from South Asian communities (Pakistani, Indian, British Asian); 3 female, 1 male, 1 undisclosed; 2 support staff (no demographic information).	UK; workshops held online, in London and Leeds	32–65	20 (13 self‐advocates, 5 carers, 2 support staff)	People with learning disabilities from ethnic minority backgrounds frequently encountered discrimination in healthcare, but it was often difficult to determine whether this was due to ethnicity, disability or both. Participants reported a lack of culturally sensitive services and reasonable adjustments, poor continuity of care, communication barriers and inadequate support during transitions. The COVID‐19 pandemic increased challenges, leading to isolation and reduced access to services. Found that digital exclusion and low awareness of the Learning Disability Register could hinder access to information. A limitation noted in the article was no participants from Jewish, asylum seeker or Traveller communities, or non‐English speakers.
7	Durling et al. (2018)	Experiences of parents with intellectual disabilities	Various community settings; recruitment through intellectual disability services and day centres	Qualitative	Semi‐structured interviews; thematic analysis	Parents with intellectual disabilities (3 mothers, 1 father); their family members; community organisation representatives‐ all from the Bangladeshi community.	London Borough of Tower Hamlets		14 (4 parents with intellectual disabilities, 4 family members, 6 community members).	Explored cultural values and practices in the experiences of parents with intellectual disabilities from the Bangladeshi community in London. The concept of “learning disability” was culturally and linguistically alien to participants. Parenting was seen as a shared responsibility across extended families. Participants emphasised that it was important for everyone to experience family roles and marriage; marriage also seen as a way to secure future support. Found tensions around differing concepts of independence, while a belief in the family's duty to care was sometimes related to family disengagement from services. Services may prioritise Western models of disability and independence without understanding cultural values, and this has implications for culturally sensitive service delivery.
8	Hatton et al. (2010)	Family carers' experiences of challenging behaviour	Various community‐based services	Qualitative	Semi‐structured interviews; interpretative phenomen‐ological analysis (IPA)	14 family carers (parents of adults with intellectual disabilities): 7 from minority ethnic communities; 7 from majority ethnic backgrounds	Urban location in Northern England	33–70	14	Explored challenging behaviour support affecting majority and minority ethnic families. There were four key themes: (1) a broad range of difficulties beyond “challenging behaviour” as defined by services; (2) relationships with local communities varied, and minority ethnic were more likely to report stigma and isolation; (3) experiences with services were often negative, especially for minority ethnic carers, who reported language barriers and a lack of culturally appropriate support; (4) relationships with the person with intellectual disabilities ranged from fulfilling to highly dependent, and could have impacts for carers' health and wellbeing. Minority ethnic families were more likely to report negative experiences with services, and these were often linked to mistrust of services. The study emphasised the need for services to understand broader social contexts for challenging behaviour, including the socio‐economic context.
9	Larkin et al. (2018)	Experiences of social care related to cultural context	Various including residential care, independent living and support in family homes	Qualitative	Semi‐structured interviews; pluralist analytical framework	32 adults with mild/moderate intellectual disabilities from minority ethnic backgrounds. Approximately 30% were Black Caribbean, 25% Pakistani, and the remainder were Indian, Bangladeshi or Mixed/Other	West Midlands	Adults	32 participants in 29 individual and group interviews	Explored the meaning of social care for adults with intellectual disabilities from minority ethnic groups. The study challenged traditional concepts of cultural competence, suggesting “cultural affordance” as a more useful framework. Participants navigated cultural resources and identities with agency. Culture was found to be context dependent. Services were evaluated positively by participants, for the most part, and were understood through relationships with particular support staff. There were a range of narratives of independence. Continuity of care and ownership of decision making were important to participants. Culturally sensitive practice requires exploration of people's cultural resources and preferences. Across cultures, good quality care develops through good relationships.
10	Malik et al. (2017)	Experiences of social care related to cultural context	Community‐based social care services including day services, supported accommodation and support in family homes	Qualitative	Semi‐structured group and individual interviews with visual elicitation tools; IPA	Opportunity sample of Women from British South Asian communities (Pakistani, Indian, Bangladeshi) with mild/moderate intellectual disabilities	West Midlands	24–48	10	Explored the role of social care services for British South Asian women with intellectual disabilities. Participants were generally satisfied with services. Services facilitated the development of complex cultural identities, allowing participants to negotiate traditional and British values (term used in source). Conflicts were found between family expectations and participants' personal aspirations for independence, which were promoted by services. Participants experienced “triple intersectionality” due to gender, ethnicity and disability, with experiences of disadvantage across all three dimensions. Tensions may arise when services ignore cultural identities. However, it was found that families were meeting most participants' cultural needs. A “good” cultural position allows people to develop their own potential, but this entails maintaining important relationships. Services need to understand people's intersectional experiences and goals related to identity.
11	Leeson and Dunstan (2023)	Experiences of and barriers to support services	Community‐based settings including supported living, residential care and family homes	Qualitative	Desktop review of prior work by the organisation; workshops and one‐to‐one conversations	People with intellectual disabilities from minority ethnic communities. 7 with intellectual disabilities (including 2 with profound and multiple intellectual disabilities); 4 autistic people; 3 with both intellectual disabilities and autism; 4 family members. Ethnicity: 7 from South Asian communities; 4 Black Caribbean; 3 Black African; 3 dual heritage participants.	UK; workshops in Birmingham and London	Adults	18	Grey literature. Report found that people with learning disabilities and autistic people from minority ethnic communities often face “double” or compounded discrimination related to the intersectionality of race and disability. Participants reported that cultural identity was neglected by services, which included being denied culturally appropriate food and a lack of support for religious practices and personal care routines, as well as a lack of diverse materials. This could impact wellbeing and lead to a loss of cultural identity and community connections. Services, communities and self‐advocacy organisations prioritised disability over other identities. Some participants felt that stereotypes about race influenced their support. Participants valued support staff who took a genuine interest in their cultural heritage and supported cross‐cultural communication. Report emphasised the need for culturally competent support that respects and reflects heritage.
12	McCarthy et al. (2021)	Forced marriage—family carers' perspectives	Community‐based care (families of people with intellectual disabilities living in family homes)	Qualitative	Focus groups and semi‐structured interviews; thematic analysis	Family carers of people with intellectual disabilities from South Asian communities; 20 women (18 mothers, 1 aunt, 1 sister), 2 men (1 father, 1 brother)	United Kingdom	Adults	22	This study found that forced marriage of people with intellectual disabilities can be motivated by family members' desire to secure long‐term care for relatives. Families experienced a burden of care, which was related to low trust in public services. Cultural and religious beliefs about marriage and disability were also found to play a role, including the idea that marriage may alleviate disability, although there were diverse views about whether family members with intellectual disabilities should marry. Views on forced marriage were changing between generations, and many participants felt that forced marriage was becoming less common in their communities. Carers were not always aware of legal concepts relating to marriage in the UK including capacity and consent, while some believed parental judgement should override legal standards. Some were distressed by formal capacity assessments while others found them helpful. Some carers resisted the idea of marriage for their disabled relatives due to fears of abuse or neglect. Others experienced community pressure for family members to be married. The study highlights the need for culturally sensitive services and provision of clear information on the law and marriage for carers.
13	McClimens et al. (2012)	Experiences of social care related to cultural context	Community‐ based settings including local intellectual disability services	Qualitative	Focus group and written responses; thematic analysis	People of Pakistani origin with a family member with intellectual disabilities (all parents)	Rotherham	Not provided	12 (8 focus group participants, 4 written responses)	This study aimed to inform cultural competence training for a learning disability/social work course through development of a simulation model, focusing on cultural needs. Participants reported inflexible and sometimes culturally insensitive services, including in education and health and social care. Language barriers were impacted by translation services that did not support some community languages, as well as general poor communication. Some reported a lack of understanding of and provision for cultural and religious needs in services such as schools, including a lack of gender‐appropriate care, poor provision of halal food and incorrect assumptions about religious practices. Found a misconception that South Asian families always provide their own care. In reality, some participants had no support from extended families. Findings led the authors to conclude that their training provision and placements should engage with more organisations and families from minority ethnic communities, to improve cultural competence and understand the diversity of minority ethnic groups.
14	Pestana (2011)	Experiences of social inclusion	Community‐based services; recruitment through third sector independent living project	Qualitative	Semi‐structured interviews; IPA	People with mild learning disabilities from minority ethnic backgrounds (Pakistani, Serbian, Irish, Indian), all living independently with support.	London	22 to 55	4	Participants reported experiences of social isolation, past abuse, and a lack of cultural support. Most participants reported that they did not receive support with their cultural needs. Some experienced discrimination in the community based on both disability and ethnicity. Most participants were employed and felt supported at and satisfied with work. The study highlights the need for service providers to ensure that minority issues are an integral part of strategic planning and staff training, so that services can help prevent social isolation and meet the cultural needs of people from minority ethnic communities.
15	Poxton et al. (2012)	Experiences of and barriers to health and social care services	Community‐based services, including statutory and third sector organisations	Qualitative	Action research: interviews and workshops	Interviews with families of children, young people and adults with intellectual disabilities from Black and minority ethnic communities (Pakistani, Bangladeshi, Indian, Ghanaian, Somali, and Chinese communities); workshops and community consultation with local projects and community leaders	Two London boroughs; one city in England outside London	Family carers of adults and child‐ren	28 families	Grey literature. This study found barriers to accessing services, including a lack of culturally competent practice, language barriers, limited understanding of complex systems and insufficient outreach to minority communities. Families described experiences of stigma, isolation and lack of trust in services. They valued continuity and culturally appropriate support. Community organisations are important in bridging gaps and supporting families to overcome barriers. Personalisation and advocacy were seen as potential solutions, but there was a need for better support to access these. The possibility of institutional racism was suggested, in relation to these systemic barriers.
16	Roy et al. (2009)	Information needs when engaging with services	Community‐based services, including some health and social care provision	Qualitative	Focus groups; thematic analysis. Collaborative research with Mencap and community organisations.	Pakistani family carers of people with intellectual disabilities	England; recruitment through groups in Dudley, Sheffield and the Midlands	Not provided	Three focus groups; participant numbers not provided	Grey literature. Families described barriers to diagnosis, stigma, difficulties managing challenging behaviour and difficulties accessing culturally appropriate support. They lacked understanding of complex social care systems. There were cultural barriers to accessing respite and planning future care. Language barriers were significant for some, affecting understanding of diagnosis and access to services. Being offered the choice to communicate in preferred languages made it easier to communicate about sensitive topics. Translation alone was not enough to overcome barriers, especially for those who could not write in their mother tongue. Trust with professionals and shared cultural context, such as in peer support groups, was a factor in understanding and acting on information about services.
17	Sandhu et al. (2017)	Experiences of Turkish‐speaking families of people with intellectual disabilities who migrated to the UK	Community‐based services; recruitment through day services for people with intellectual disabilities	Qualitative	Semi‐structured interviews; narrative analysis	Turkish‐speaking migrant families (from Kurdish, Turkish Cypriot and mainland Turkish backgrounds) with a child with intellectual disabilities. Four families were from one city borough with a large Turkish‐speaking population; a fifth family were from another borough in the same city	City in the UK	Families with children aged 10 to 39	Five families	This study found that the care and other needs of family members with intellectual disabilities were often central to migration decisions, together with the impact of geopolitical conflicts. Families described scarce or poor services in their countries of origin. The UK was seen as an idealised location that could offer improved support and opportunities for their family members, but post‐migration experiences often fell short of expectations. Families reported disempowerment, communication difficulties with services, and concerns that children would be removed from their care following interactions with staff. Some families disengaged from services due to these challenges. Most expressed gratitude for services, buy many were worried for their family members' future. Families had little agency in migration decisions that had significantly affected their lives and those of their children.
18	Southby (2017)	Barriers to respite care	Community‐based respite care services (non‐residential)	Mixed methods	Postal survey, semi‐structured interviews with carers and stakeholders; consultation event with service users	Carers of adults with intellectual disabilities and/or autism with moderate to complex needs; stakeholders from adult social care and third sector organisations. Aggregated survey findings from families from minority ethnic communities on one question (11 out of 127 survey respondents were from Black, Asian and other non‐White British backgrounds); no demographic breakdown of interviews.	One northern city in England	Adults caring for people aged 18–60+	127 survey responses; 15 interviews (8 stakeholders, 7 carers); ~50 consultation event participants	This study explored actual and perceived barriers to accessing non‐residential respite care for adults with intellectual disabilities and autistic people. Carers valued respite support, but they were concerned about engaging with unfamiliar non‐residential services when they were more familiar with residential respite provision. Barriers included the fear of losing residential provision and lack of knowledge of services. There were also concerns about inflexibility, bureaucracy, adequacy of care and privacy in the home. In contrast with other published research, 91% of minority ethnic respondents said culture and religion were not a barrier to accessing non‐residential respite services; 9% said these were a barrier. This finding may have been impacted by response bias, given the limited number of respondents from minority ethnic communities (*n* = 11). There was no analysis of other barriers by ethnicity.
19	Udonsi (2022)	Barriers to health and social care	Health and social care	Qualitative	Case study; critical intersectional analysis	Case study of young Black African autistic man with an intellectual disability (second‐generation migrant)	United Kingdom	18	1	This critical analysis explores intersectional experiences of race and intellectual disability in the context of UK health and social care for an 18‐year‐old Black African man. The participant experienced culturally insensitive care, including the use of a different name rather than his own African name by staff. Other issues included long waiting lists for assessments, a lack of specialist training for support workers, and medication used to manage behaviour without considering environmental barriers. Individual budgets were challenging for the participant and his family to manage, in contrast with the assumption that personalisation improves outcomes. The young man was eventually detained in a mental health hospital. These experiences of social care were set in the context of the myth that Black African families “look after their own”. It was argued that racism, disablism and neoliberal agendas in service provision can increase the risk of incarceration for Black people with intellectual disabilities. A therapeutic approach from services in managing distress was recommended, together with anti‐racist provision to address structural barriers and systemic racism in services.

We used narrative synthesis to identify common themes and gaps in the sparse and diverse evidence, in relation to the research question (Popay et al. 2006). Sources were imported into NVivo software. After initial immersion in the literature, a preliminary coding framework was developed by the first reviewer, using an open coding process with 20% of the articles. The second reviewer then independently coded a sample of 20% of articles to validate the coding framework. The full review team developed the coding framework through discussion. The first reviewer then undertook analysis, initially in NVivo, using and building on the initial coding framework to generate themes (see Table 2, ‘Themes’), and finally through a written narrative. Analysis focused on experiences of social care, including reported structural barriers when accessing care systems. Given the range of topics represented in the data, we also examined related issues, including the ways families and people with intellectual disabilities navigated cultural values and service expectations, and their relationships with service providers and staff.

**TABLE 2:** Thematic framework used to analyse literature on social care for adults with intellectual disabilities from minority ethnic communities.

Theme 1: Access to and quality of appropriate services	
Sub‐theme	Additional codes
Availability and quality of support	Inflexibility, timing and inconsistency
	The struggle to access services
	Limited choice and control
	External barriers
Navigating services	Knowledge of services and referrals
	(Dis)connections between services
	Moving between adult and child services
Language, communication and accessibility barriers	Language barriers
	Communication with professionals and services
	Accessibility barriers
Discrimination and institutional barriers to social care	Experiences of discrimination and racism
	Mistrust of services

Theme 2: Cultural factors in social care	
Sub‐theme	Additional codes
Cultural expectations of collective family participation	(Limited) extended family support
	High “burden” of care
	Diversity in family values/expectations of family
Role of wider community	Community support
	Isolation and stigma
Concerns about future (culturally appropriate) care provision	Low expectations for future support
	Marriage as care arrangement
Impact of diverse cultural and religious values for social care	Tensions arising from services' central values (including differing concepts of independence)
	Impact of diverse concepts of/beliefs about intellectual disability
	Negotiating cultural and religious identities
(Lack of) culturally inclusive/targeted services	Mismatch of service provision and cultural/religious values
	High‐quality services not “special services”
Inclusive services and support for minority ethnic communities	Importance of support for religious beliefs/practices from services
	Services (not) facilitating community and religious participation
	Religious/cultural practices (not) supported by services
	Cultural/religious needs met by families
Intersectionality and social care	Double discrimination/multiple disadvantage
	Recognising intersectional experiences
	Socioeconomic factors in inequality
Human rights abuses and safeguarding concerns	Vulnerability to/risks of forced marriage
	Prevention of forced marriage

Theme 3: Improving cultural competency in service planning, commissioning and delivery	
Sub‐theme	Additional codes
Recommendations for policy and practice	Staff training in cultural competency
	Commissioning, planning and delivering culturally appropriate services
	Addressing discrimination, institutional racism and systemic barriers
	Monitoring service use
	Reviewing local and national policies and practice guidance
Person‐ and family‐centred culturally competent care	Continuity of care and relational support
	Involving the whole family in care provision
	Curiosity and cultural sensitivity from staff
Understanding local communities	Building networks and community partnerships with trusted organisations
	Recruiting diverse support teams
	Improving language/communication support
	Advocacy and support to navigate systems

There was no comprehensive risk of bias assessment of sources, as this was not appropriate for a rapid review synthesising a range of types of evidence (Cavanagh et al. 2024). The methodological and other limitations of each included study were recorded and considered in narrative synthesis.

## 3 Results

### 3.1 Study Selection

We identified 657 records from academic databases (*n* = 619) and additional sources (*n* = 38). Following deduplication, 540 records remained for abstract screening. Of these, 382 records were excluded, leaving 158 studies for full‐text screening. After full‐text screening, 139 studies were excluded, leaving 19 studies which met inclusion criteria (see Figure 1).

### 3.2 Study Characteristics and Overview of the Evidence Base

The included studies (see Table 1 for details) were published and distributed relatively evenly between 2009 and 2025, with no obvious increase in research volume over time. Most of the studies were conducted in urban areas with established minority ethnic populations, including London, the West Midlands, and cities in northern England (*n* = 13). Most adopted qualitative research designs (*n* = 15) and had small sample sizes, with 15 studies including fewer than 50 participants; seven had 15 or fewer participants (Udonsi 2022; Pestana 2011; Sandhu et al. 2017; Malik et al. 2017; McClimens et al. 2012; Hatton et al. 2010; Durling et al. 2018). The two studies with the largest samples were survey‐based or secondary data analysis studies with quantitative components (Clawson et al. 2020; Clawson and Fyson 2017). One more survey‐based study included 127 survey respondents (Southby 2017), but only 11 were from minority ethnic backgrounds.

Studies most commonly focused solely or largely on South Asian^3^ communities (*n* = 9) (Malik et al. 2017; McClimens et al. 2012; Durling et al. 2018; Clawson et al. 2020; Clawson and Fyson 2017; Ali et al. 2013; Bhardwaj et al. 2018; McCarthy et al. 2021; Roy et al. 2009), with a further five studies including a more diverse range of minority ethnic participants (Leeson and Dunstan 2023; Poxton et al. 2012; Bruun et al. 2025; Cooper‐Moss et al. 2025; Larkin et al. 2018). There was smaller representation of Chinese (Poxton et al. 2012; Cooper‐Moss et al. 2025), dual heritage (Leeson and Dunstan 2023; Pestana 2011; Bruun et al. 2025; Larkin et al. 2018), and individual Serbian and Irish participants (Pestana 2011). One study included Turkish‐speaking migrant communities (Sandhu et al. 2017). There were no studies focusing on Jewish or Gypsy, Roma or Traveller (GRT) communities. Some studies reported difficulties in recruiting minority ethnic participants in studies which also included majority participants (Hatton et al. 2010; Bruun et al. 2025); others noted that they did not capture the perspectives of more marginalised groups within minority communities, such as non‐English speakers (Bruun et al. 2025; Cooper‐Moss et al. 2025).

Six studies examined systemic barriers facing minority ethnic communities in accessing health and social care services for people with intellectual disabilities (Leeson and Dunstan 2023; Poxton et al. 2012; Udonsi 2022; Southby 2017; Ali et al. 2013; Cooper‐Moss et al. 2025), although two of these focused on healthcare with limited findings on social care (Ali et al. 2013; Cooper‐Moss et al. 2025). Four explored experiences of social care in relation to cultural context (Pestana 2011; Malik et al. 2017; McClimens et al. 2012; Larkin et al. 2018). Three explored forced marriage as a safeguarding concern for this population (Clawson et al. 2020; Clawson and Fyson 2017; McCarthy et al. 2021). Single studies researched social networks (Bhardwaj et al. 2018), end‐of‐life care planning (Bruun et al. 2025), experiences of parents with intellectual disabilities (Durling et al. 2018), families' information needs (Roy et al. 2009) and experiences of migrant families (Sandhu et al. 2017). Barriers to services were emerging themes in several of these studies (Hatton et al. 2010; Roy et al. 2009; Bruun et al. 2025).

Three sources were included from the grey literature search. These consisted of primary research by community and policy groups: Mencap (Roy et al. 2009), the Foundation for People with Learning Disabilities (Poxton et al. 2012), and Changing Our Lives (Leeson and Dunstan 2023). Although these studies shared more limited findings than the academic sources, they were included due to the scarcity of primary academic research on the topic and to broaden the range of the review by including community‐focused research perspectives.

#### 3.2.1 Research Involvement of People With Intellectual Disabilities and Their Families

Eleven studies included research participants with intellectual disabilities (Leeson and Dunstan 2023; Udonsi 2022; Pestana 2011; Malik et al. 2017; Durling et al. 2018; Southby 2017; Ali et al. 2013; Bhardwaj et al. 2018; Bruun et al. 2025; Cooper‐Moss et al. 2025; Larkin et al. 2018). Only one study included participants with profound or multiple intellectual disabilities (Leeson and Dunstan 2023); four studies explicitly excluded this population (Pestana 2011; Ali et al. 2013; Bhardwaj et al. 2018; Bruun et al. 2025). Eight studies did not include direct participation from people with intellectual disabilities; six of these focused solely on family carer perspectives (Poxton et al. 2012; Sandhu et al. 2017; McClimens et al. 2012; Hatton et al. 2010; McCarthy et al. 2021; Roy et al. 2009) and two analysed interviews or secondary data from professionals and services (Clawson et al. 2020; Clawson and Fyson 2017). The three studies on forced marriage drew on professional or family carer accounts, without involvement from people with intellectual disabilities (Clawson et al. 2020; Clawson and Fyson 2017; McCarthy et al. 2021). All three grey studies included people with intellectual disabilities and/or their families (Leeson and Dunstan 2023; Poxton et al. 2012; Roy et al. 2009).

### 3.3 Themes

The literature was organised into three themes: (1) Access to and quality of appropriate social care services; (2) Cultural factors in engagement with social care; (3) Cultural competence in service planning, commissioning and delivery, broken down into further sub‐themes for detailed analysis (see Table 2).

Theme 1 Access to and quality of appropriate social care services.

Several studies found that people with intellectual disabilities and their families from minority ethnic communities face similar barriers to accessing social care as people from majority communities, but that these are compounded by additional intersectional disadvantages (Udonsi 2022; Hatton et al. 2010; Roy et al. 2009; Larkin et al. 2018). Reported obstacles related to the sufficiency, flexibility, timing, inconsistency and ineffectiveness of service provision for people's needs (Poxton et al. 2012; McClimens et al. 2012; Hatton et al. 2010; Ali et al. 2013; Roy et al. 2009; Cooper‐Moss et al. 2025). Accessing services often required struggle and perseverance, as families spoke of having to battle the system to secure appropriate support (McClimens et al. 2012; Hatton et al. 2010; Ali et al. 2013; Roy et al. 2009), in a climate of fear of services being withdrawn (Larkin et al. 2018).

Knowledge of services and referrals were particular barriers to navigating social care provision. Participants with intellectual disabilities and their families reported low awareness of services and a lack of information about them (Poxton et al. 2012; Hatton et al. 2010; Ali et al. 2013; Roy et al. 2009; Cooper‐Moss et al. 2025). Finding out about complex services could involve labour for families, and was sometimes dependent on familiarity with systems and connections with individuals, rather than simple eligibility (Leeson and Dunstan 2023; McClimens et al. 2012; Ali et al. 2013; Cooper‐Moss et al. 2025). Several studies discussed disconnections between services and unclear referral pathways (Poxton et al. 2012; Ali et al. 2013; Roy et al. 2009; Cooper‐Moss et al. 2025), particularly when families were moving from child to adult services. While moving from child to adult services can be challenging for people with intellectual disabilities and their families from all communities (Pallisera et al. 2016), these were exacerbated for participants from minority ethnic communities, who may particularly struggle to navigate complex services that do not “present a clear… ‘front door’” (Poxton et al. 2012), p. 6.

There was evidence that services were not always offering adequate choice, control or support to participants. In one study, participants with intellectual disabilities reported that they valued the social care services they received, especially for social connectedness, but had limited involvement in decision‐making about service changes or withdrawal (Larkin et al. 2018). In contrast to the general perception that personal budgets improve access to social care, some studies noted barriers to navigating the personalisation system, including insufficient advocacy and information (Poxton et al. 2012; Roy et al. 2009) and difficulties finding appropriate carer support when using direct payments (Udonsi 2022; McClimens et al. 2012).

Language and communication barriers were reported in a number of studies, including but not limited to translation services. Participants had varying needs for interpreters and translation, and services were not always responsive to the translation needs of specific communities (Leeson and Dunstan 2023; Poxton et al. 2012; McClimens et al. 2012). However, while interpreters could improve communication with professionals, they could also impede effective communication (Sandhu et al. 2017).

More generally, the complex terminology used in social care systems and professional language was a barrier for those with poorer communicative ability or for whom English was a second language (Hatton et al. 2010; Ali et al. 2013; Bhardwaj et al. 2018; Roy et al. 2009; Cooper‐Moss et al. 2025). Poor communication by professionals compounded other barriers, sometimes leading participants to disengage from services (Sandhu et al. 2017; McClimens et al. 2012; Hatton et al. 2010; Roy et al. 2009) or feeling too intimidated to question professionals' decisions (Poxton et al. 2012). Conversely, offering communication in preferred languages could improve experiences of services (Leeson and Dunstan 2023; Hatton et al. 2010; Roy et al. 2009; Cooper‐Moss et al. 2025). In a few studies, language barriers were exacerbated by accessibility issues such as poor availability of Easy Read or inaccessibility of digital resources (Leeson and Dunstan 2023; Poxton et al. 2012; Ali et al. 2013; Cooper‐Moss et al. 2025).

There was also evidence of discrimination and institutional barriers in services. Racism and the perception of racism in social care were particularly discussed within the grey literature, among other studies, often in relation to relationships with professionals and the importance of these to the quality of care received (Leeson and Dunstan 2023; Poxton et al. 2012; Udonsi 2022; Roy et al. 2009; Cooper‐Moss et al. 2025). Leeson and Dunstan (Leeson and Dunstan 2023) reported participants' concerns about poor treatment and discrimination for Black family members with intellectual disabilities, including the use of Anglicised rather than given names, an issue also discussed by Udonsi (Udonsi 2022). Experiences of service shortcomings were implicated in ongoing mistrust of and disengagement from services in several studies (Poxton et al. 2012; Hatton et al. 2010; Clawson et al. 2020; McCarthy et al. 2021). Poxton et al. argued that culturally insensitive services may be perceived as “racist rather than lacking understanding” (Poxton et al. 2012), p. 11, and linked mistrust of services to poor cultural competence and high staff turnover.

Theme 2 Cultural factors in Engagement with Social Care.

There was a focus on cultural expectations and family roles in South Asian communities in several studies (Malik et al. 2017; Durling et al. 2018; Bhardwaj et al. 2018; McCarthy et al. 2021; Larkin et al. 2018), with some exploration of cultural particularities in the role of extended families in specific minority ethnic communities. Durling, Chinn and Scior (Durling et al. 2018) explored a collective approach to family life in the Bangladeshi community and its implications for parents with intellectual disabilities. They considered the “duty to care” to be a fundamental cultural value, associating this with Muslim religious beliefs and arranged marriages. McCarthy et al. (McCarthy et al. 2021) identified a similar cultural duty to provide care as a potential motivator for forced marriage, while marriage—arranged or forced—was discussed as a perceived solution to the problem of securing future care for family members with intellectual disabilities (Durling et al. 2018; Clawson et al. 2020; Clawson and Fyson 2017; McCarthy et al. 2021). Participants in some other studies also discussed cultural values and expectations around family care (Poxton et al. 2012; Roy et al. 2009). However, there were also reports of broader structural inequalities which left participants reliant on extended family support. Shared family care could be a response to inadequate service provision (Poxton et al. 2012; Sandhu et al. 2017), while cultural expectations that families should provide care could lead to a reluctance to engage with services (McClimens et al. 2012; Roy et al. 2009).

Furthermore, for participants in several studies, the care support offered by extended families was limited or non‐existent (Sandhu et al. 2017; Malik et al. 2017; McClimens et al. 2012; Durling et al. 2018; Ali et al. 2013; Bhardwaj et al. 2018; McCarthy et al. 2021; Cooper‐Moss et al. 2025), and some studies specifically pointed out that South Asian family carers did not have the supportive extended family structure that is traditionally expected of this community (McClimens et al. 2012; Cooper‐Moss et al. 2025). A high “burden of care” (McCarthy et al. 2021), p. 204 for families from minority ethnic communities was identified in some studies (Malik et al. 2017; Durling et al. 2018; Clawson and Fyson 2017; McCarthy et al. 2021; Roy et al. 2009), sometimes related to a lack of appropriate support to access services (Poxton et al. 2012; Roy et al. 2009) or low trust in services (McCarthy et al. 2021). Some studies related this to the myth that minority ethnic communities “look after their own,” and service providers' perception that services are unnecessary because the extended family or community will provide care (Udonsi 2022; McClimens et al. 2012; McCarthy et al. 2021).

Cultural and religious identities and values were explored in a number of studies, including values attributed by researchers to minority ethnic communities' cultures and/or religions, and their impact on the takeup or experiences of statutory services (Leeson and Dunstan 2023; Poxton et al. 2012; Pestana 2011; Malik et al. 2017; Durling et al. 2018; McCarthy et al. 2021; Roy et al. 2009; Bruun et al. 2025; Larkin et al. 2018). Some studies explored the meaning of *culture*, finding that cultural and religious commitments and practice were context‐dependent and actively negotiated by people with intellectual disabilities (Malik et al. 2017; Larkin et al. 2018), and arguing that there is a need for service providers to support cultural choices and facilitate the development of complex cultural identities. Leeson and Dunstan (Leeson and Dunstan 2023) reached similar conclusions.

Some studies explored tensions between service providers and families over differing values, such as expectations of family roles or the Western concept of independence which are central to UK service provision (Malik et al. 2017; Durling et al. 2018; Clawson and Fyson 2017; Bruun et al. 2025; Cooper‐Moss et al. 2025; Larkin et al. 2018). Other studies found that cultural values and religious beliefs, including varying cultural concepts of disability, could affect whether some participants sought support from services (Poxton et al. 2012; Malik et al. 2017; Durling et al. 2018; McCarthy et al. 2021; Roy et al. 2009). However, perceptions of clashing values may be based on assumptions made by staff (Bruun et al. 2025), and concepts of “culture clash” may be too simplistic to explain differences in priorities between families and service providers (Malik et al. 2017).

The issue of inclusive services for people from minority ethnic communities, and what these might consist of, arose in many of the studies. Some studies reported evidence of mismatches between families' cultural and religious needs and how far services supported these, for example gender separation in provision of care (Poxton et al. 2012; Malik et al. 2017; McCarthy et al. 2021; Roy et al. 2009; Cooper‐Moss et al. 2025). In a few studies, specialist services targeted to meet the needs of particular communities were associated with higher levels of satisfaction and engagement with services (Malik et al. 2017; Durling et al. 2018). However, the grey studies noted that minority ethnic families value high‐quality services over “special services” (Leeson and Dunstan 2023; Poxton et al. 2012; Roy et al. 2009); one concluded that participants did not always want the services that professionals may “think they should want” (Poxton et al. 2012), p. 10.

The importance of religious practices and cultural and community participation for participants' identities and ways of life was noted in several studies (Leeson and Dunstan 2023; Poxton et al. 2012; Pestana 2011; Malik et al. 2017; Bruun et al. 2025; Larkin et al. 2018). However, participants in a few studies reported that services often failed to accommodate these needs. Leeson and Dunstan (Leeson and Dunstan 2023) found that faith practices and cultural preferences were poorly respected in residential care settings, for example, the provision of proscribed food, limited interest from care staff in people's cultural or ethnic backgrounds, a lack of diverse resources and variable levels of support to connect with their communities; many of these issues were also identified in Pestana's study (Pestana 2011). A few studies more positively found that care staff had supported people to continue Christian and Muslim religious practices and maintain cultural and community connections (Leeson and Dunstan 2023; Bruun et al. 2025). One study argued that cultural and religious needs may be more effectively facilitated by family rather than services (Malik et al. 2017), although two participants in this study who did not live in family homes found that their religious practice and language were less well supported by services.

A few studies addressed intersectionality in the context of inequalities in social care. One study found that, while participants did not report direct discrimination by services, they experienced intersectional disadvantage as South Asian women with intellectual disabilities (Malik et al. 2017). However, the study primarily explored culture and family as barriers to opportunity, rather than examining external systemic barriers to social care or social participation. Pestana's research (Pestana 2011) also reflected intersectionality, with participants reporting that social isolation and mistreatment from the wider community could occur in response to either ethnicity or intellectual disabilities. Leeson and Dunstan discussed “double discrimination (at the intersection of disability and race)” (Leeson and Dunstan 2023), p. 13, and argued that while statutory services may recognise disability, people's racial, cultural and religious identities are less well recognised or supported. In systemic terms, Udonsi (Udonsi 2022) critiqued care services that erase intersectional experiences for Black people, and argued for the importance of anti‐racist services, as did Leeson and Dunstan (Leeson and Dunstan 2023). Another study considered broader socioeconomic inequality as part of the full picture of inequality for people from minority ethnic communities (Hatton et al. 2010).

Three studies focused on human rights abuses in the context of forced marriage (Clawson et al. 2020; Clawson and Fyson 2017; McCarthy et al. 2021), finding that people with intellectual disabilities are more vulnerable to forced marriage than other demographics. This vulnerability was discussed in relation to cultural and religious beliefs and values, including beliefs about intellectual disabilities (McCarthy et al. 2021) and concepts of consent (Durling et al. 2018; Clawson and Fyson 2017). Social care professionals were found to play a key role in identifying and preventing forced marriage (Clawson et al. 2020; Clawson and Fyson 2017).

Theme 3 Improving cultural competence in service planning, commissioning and delivery.

There were several recommendations for improvements to social care provision for people with intellectual disabilities from minority ethnic communities and their families, with some focus on cultural competence.^4^

- Recommendations for policy and practice

Several studies recommended cultural competence training for staff (Poxton et al. 2012; Pestana 2011; Clawson et al. 2020; Clawson and Fyson 2017; Bruun et al. 2025), to improve understanding of different cultures and religions (Poxton et al. 2012; Bruun et al. 2025), encourage staff knowledge and interest in people's ethnic and cultural backgrounds (Leeson and Dunstan 2023), and support trust‐building and signposting to services (Poxton et al. 2012; Roy et al. 2009). Literature on forced marriage noted the need to raise awareness among social care professionals about the safeguarding risks of forced marriage (Clawson et al. 2020; Clawson and Fyson 2017; McCarthy et al. 2021). A few studies argued that training should address professional misconceptions about extended family support in minority ethnic communities, given that the myth that communities “look after their own” may result in reduced provision and lower engagement with services (McClimens et al. 2012; Ali et al. 2013).

At a policy level, a few studies emphasised that services should be adequately resourced to ensure that people can be helped to navigate complex systems such as personalisation and to allow for efforts to identify and support marginalised groups such as older carers (Poxton et al. 2012; Udonsi 2022). Recommendations for commissioners included taking ethnicity and intersectionality into account when planning and tendering for service provision, commissioning culturally appropriate services (Leeson and Dunstan 2023; Poxton et al. 2012; Udonsi 2022; Roy et al. 2009), and translation and interpretation services targeted to community language needs (Poxton et al. 2012; McClimens et al. 2012; Roy et al. 2009). Some studies advocated for culturally competent practice, including supporting people to attend religious services, follow religious and cultural food laws and preferences, and participate in cultural and community activities (Leeson and Dunstan 2023; Pestana 2011; Bruun et al. 2025), and gender‐matching staff when requested (Poxton et al. 2012; Malik et al. 2017; McCarthy et al. 2021; Roy et al. 2009; Cooper‐Moss et al. 2025). A few studies encouraged open discussion about cultural and religious practices in care planning (Leeson and Dunstan 2023; Bruun et al. 2025; Cooper‐Moss et al. 2025). Others recommended that providers monitor service access by minority ethnic communities and review policies for potential institutional racism (Poxton et al. 2012; Udonsi 2022). Studies on forced marriage called for the inclusion of this issue in UK safeguarding guidance (Clawson et al. 2020) and other policy and practice guidance (McCarthy et al. 2021). Others argued that services need to better understand the culturally specific and varied ways in which minority communities may understand concepts such as “intellectual disabilities” and “independence” (Malik et al. 2017; Durling et al. 2018).

b Person‐ and family‐centred approaches to culturally competent care

In several studies, cultural competence is shown to be person‐centred, supported by positive relationships and continuity of care (Leeson and Dunstan 2023; Poxton et al. 2012; Bruun et al. 2025; Cooper‐Moss et al. 2025; Larkin et al. 2018). In this context, some studies cautioned against assumptions about care or service needs based on stereotyped or simplistic understandings of ethnicity or religion (Leeson and Dunstan 2023; Poxton et al. 2012; Malik et al. 2017; Bruun et al. 2025; Larkin et al. 2018). One study argued that service providers, entering people's homes as outsiders, must develop culturally sensitive understanding through curiosity about those they support (Larkin et al. 2018). Another considered curiosity about people's cultures, ethnicities and religions to be central to culturally competent care (Leeson and Dunstan 2023).

Relatedly, a few studies found that involving the whole family in care planning could enhance aspects of culturally competent care (Leeson and Dunstan 2023; Bruun et al. 2025). Some of the grey literature recommended that care providers engage with wider families, including siblings, who are often involved in translation and other shared care (Poxton et al. 2012; Roy et al. 2009); one of the grey studies also recommended training to help practitioners understand and support whole families (Roy et al. 2009). Some of the studies that focused on forced marriage argued for more culturally competent care and improved access to services, to encourage families' engagement with social care, thereby reducing this safeguarding risk (Clawson et al. 2020; McCarthy et al. 2021).

c Understanding local communities

Several studies encouraged commissioners and providers to build networks with trusted community and religious organisations to deepen understanding of local contexts beyond homogenous notions of ethnicity (Poxton et al. 2012; McClimens et al. 2012; Roy et al. 2009; Bruun et al. 2025). Some of the grey studies recommended investing in community organisations already trusted by minority ethnic communities, through commissioning, for example (Poxton et al. 2012). Leeson and Dunstan recommended recruiting diverse staff teams that reflect the communities served, but cautioned against assuming everyone from minority ethnic backgrounds wishes to be supported by care staff of the same ethnicity (Leeson and Dunstan 2023), p. 18.

There were some recommendations for improving communication, including establishing priorities for languages for translation and other targeted communication, through an understanding of local communities (Poxton et al. 2012; McClimens et al. 2012). Roy and colleagues (Roy et al. 2009) noted that many first‐generation South Asian immigrants cannot read and write in their first language, making translated materials of limited use; audio‐visual resources were suggested instead. Leeson and Dunstan (Leeson and Dunstan 2023) found that it could help to build trust if staff spoke their languages or had an understanding of the role of different languages in participants' cultural contexts. Clear communication about cultural and religious preferences in end‐of‐life care, as part of person‐centred planning, was recommended in one study (Bruun et al. 2025).

Several studies, including the grey literature, identified a lack of culturally sensitive and inclusive self‐advocacy services (Leeson and Dunstan 2023; Poxton et al. 2012; Roy et al. 2009; Cooper‐Moss et al. 2025). The self‐advocacy movement was also encouraged to take an intersectional, anti‐racist approach, led by senior management (Leeson and Dunstan 2023; Udonsi 2022).

## 4 Discussion

This rapid review found sparse research evidence on the social care experiences of adults with intellectual disabilities from minority ethnic communities in the UK. With only 19 studies distributed relatively evenly across the 15‐year range, we found no clear sign of increasing research on this topic. The extant research also consists primarily of small studies. While small samples are appropriate for the exploratory qualitative research that represents most of this evidence, there is a limited range of research designs across these sources. Together, these characteristics suggest an underdeveloped field of research.

Findings of this review indicate that people with intellectual disabilities from minority ethnic communities face similar challenges to those from majority ethnic communities, but that these challenges are compounded by additional intersectional disadvantages. Complex social care services are shown to be difficult to navigate, which can lead to difficulties accessing services, with examples of language barriers and inadequate communication from service providers. There are reports of experiences of racism for those who engage with services, and perceptions of racism when services provide inadequate or insufficient care, related to mistrust and avoidance of engagement with services. There was some evidence of services that do not meet people's cultural, ethnic and religious needs. A lack of support for family carers is in evidence, with suggestions that the stereotype that people from minority ethnic communities “look after their own” (Katbamna et al. 2004) continues to influence some services' responses to the need for support for carers; this may have an impact on services' ability to address safeguarding issues, such as forced marriage. These findings reflect earlier research and other literature reviews, which have identified similar systemic barriers to accessing and navigating social care for this population (Caton et al. 2007; Azmi et al. 1997; Devapriam et al. 2008; Mir et al. 2001; Laird 2008; Poxton et al. 2012; Hatton et al. 1998; Wilkinson 2009; Tawodzera et al. 2025; Raghavan 2009).

However, our review found limited academic research investigating how far and how successfully services provide culturally competent care. There was little research exploring barriers to accessing social care services, and limited discussion of systemic institutional disablism, which Hatton has defined as “the collective failure of organisations to provide an appropriate and professional service to people because of their [intellectual disability]” (Hatton 2026), or institutional racism in services (Burke and Ong 2021; Mir et al. 2001; Laird 2008). Only a few studies argued for the importance of anti‐racist services, or services that tackle racism (Leeson and Dunstan 2023; Udonsi 2022), although this has been noted in broader critical race scholarship on social care for people from minority ethnic communities and in policy advocacy on social care for this population (Butt and Mirza 1996; Burke and Ong 2021; Baxter et al. 1990; Mir et al. 2001; Laird 2008). There was minimal research that used a critical race theory lens to explore inequalities in social care; one exception (Udonsi 2022) critiqued a neoliberal social care agenda that may overlook intersectional experiences for Black people with intellectual disabilities, increasing risks, including misdiagnosis and unnecessary detention.

There is also an imbalance in demographic focus across these studies, with some skew towards South Asian communities, particularly in the studies focused on specific communities. This focus reflects the UK's historical migration patterns: people from South Asian communities together make up the largest minority ethnic group in England and Wales (Office of National Statistics (ONS) 2021). However, the imbalance leaves other minority ethnic communities' social care experiences underexplored. Robertson and colleagues (Robertson et al. 2019) found a similar focus on South Asian communities in their systematic review of health care for people with intellectual disabilities from minority ethnic communities. There is a notable lack of research focus on other communities; for example, the lack of GRT inclusion is concerning given that GRT communities are known to experience particularly poor health outcomes and low trust with health and social care providers (Turbett and Pye 2024; McFadden et al. 2018). There is a noted lack of trust in health and social care research from Black communities (Corbie‐Smith et al. 2002; Farooqi et al. 2022), which may account for some of the lack of demographic focus on Black communities in this evidence, although local community interests and university or funder research agendas may also play a role. Nonetheless, this imbalance should be seen in the context of insufficient research on health and social care for all minority ethnic communities, compared with white populations.

There were other under‐represented perspectives across these studies. Only one study explicitly included people with profound and multiple intellectual disabilities (Leeson and Dunstan 2023), suggesting that their perspectives and their families are less well explored; this reflects the lack of inclusion of this group in research on other topics (de Haas et al. 2022). Furthermore, many studies recruited through statutory services (Pestana 2011; Sandhu et al. 2017; Malik et al. 2017; Durling et al. 2018; Bhardwaj et al. 2018), which may be an oversight given the low service takeup and potential mistrust of services noted in several of the studies. More of a range of recruitment methods may be needed to reach people who do not engage with services.

There is also some imbalance in topic focus across this research. Three out of 19 studies focused on forced marriage, primarily in South Asian communities (Clawson et al. 2020; Clawson and Fyson 2017; McCarthy et al. 2021), reflecting findings that most forced marriages of people with intellectual disabilities in the UK occur among South Asian communities (Clawson et al. 2020). These studies explore and make recommendations on a significant safeguarding issue. However, Gill and Anitha (Gill and Anitha 2011) argue that a concern about forced marriage which concentrates on South Asian communities may be stigmatising for people from these communities, while a focus on consent may minimise people's agency in complex situations. In the absence of other research, an overemphasis on this topic may result in an underestimation of systemic barriers affecting South Asian and other minority communities' access to appropriate social care, as well as other safeguarding risks.

Several of the academic studies positioned cultural differences as barriers to service engagement, in research looking at the impact of minority ethnic participants' beliefs, values and practices for access to and provision of care. Culture was not always clearly defined in these studies and was sometimes conflated with religion. It is not always useful to think of culture and religion as discrete categories, since they may be closely connected in the lives of people from minority ethnic communities who are supported by social care services (Larkin et al. 2018; Hordern 2016). However, Ahmad and Atkin have argued that social care services may pathologise cultural differences among people from minority ethnic communities (Ahmad and Atkin 1996). In this context, an unclear framing of ‘culture’ may position minority ethnic communities and their beliefs and practices as problems to be solved (Mir 2004). For example, several studies focused on differences between the Western values of services, which are likely to emphasise independence, and minority communities' contrasting perspectives on interdependence and family roles. These studies demonstrate potential differences in understanding of concepts such as independence or intellectual disabilities which are useful for professionals to appreciate. However, such differences in viewpoint were sometimes represented as cultural “tensions” (Durling et al. 2018) or problems, without problematising the Western‐centric values of providers in the same way. There is also a strong focus across the research on cultural values and family roles in South Asian communities in particular. Although the myth that minority ethnic families “look after their own” (Katbamna et al. 2004) was challenged in some studies (Udonsi 2022; McClimens et al. 2012; Ali et al. 2013), the focus on culturally mediated family values may suggest that such stereotyping persists. In reality, there were reports of limited extended family support from participants in several studies, while other studies suggested that shared family care could be a response to inadequate service provision rather than a cultural choice.

Additionally, the focus on cultural differences may risk obscuring structural barriers implicated in the low trust between families and service providers noted in some of these sources. While some studies found that people with intellectual disabilities actively negotiate complex cultural identities (Malik et al. 2017; Larkin et al. 2018), others reported that social care services failed to facilitate their cultural needs and religious participation (Leeson and Dunstan 2023; Pestana 2011). There was some evidence of services that see people only through the lens of one “master status” (Goffman 1963)—usually as people with intellectual disabilities—rather than supporting the full complexity of people's intersectional identities (Crenshaw 1989). However, while intersectionality was a focus of a few studies, overall there is limited research attention paid here to intersectional and broader socioeconomic factors in the context of social care. This constrains understanding of the ways that multiple disadvantage can impact people's access to appropriate support (Udonsi 2022; Hatton et al. 2010).

Notably, the three grey studies focused more directly on barriers to accessing services and inadequate cultural competence in services, including variable support of people's cultural needs and religious practices (Leeson and Dunstan 2023; Poxton et al. 2012; Roy et al. 2009). Given their practical and policy focus, these studies were the source of many of the recommendations for providers discussed above. All three studies also had direct participation from people with intellectual disabilities and their families, which was not always the case in the academic literature. These differences in focus and methods may suggest a disjuncture between community research priorities and academic research in this area.

The recommendations emerging from this review, particularly in the grey literature, highlight the need for systemic change in social care provision, including the importance of addressing institutional racism, resourcing services adequately, and supporting individuals and families to navigate complex systems. Across much of the research, there is a recognition that services should afford people opportunities and agency to navigate their own intersectional identities and cultures, as well as engaging with curiosity and interest in people's cultural backgrounds and religious practices. A number of studies note the importance of understanding local populations of people who engage with social care services, or who may engage with them, with some pointing out that communities may not always value the services that commissioners and providers think they need. This requires a knowledge of local communities from service providers that goes beyond homogenising and stereotyping. This is likely to entail a need for more detailed data than is currently collected on this population (Umpleby et al. 2023; Burke and Ong 2021). However, more research is needed to explore how far the barriers and recommendations reflected in some of this evidence will be borne out by larger‐scale research, including quantitative studies.

## 5 Conclusion

The limited evidence represented in this rapid review has implications for future research, policy and practice. The focus on certain communities and topics, while important, does not represent a diverse range of research into how effectively social care services are supporting people with intellectual disabilities from a range of minority ethnic communities. Furthermore, the lack of research with people with profound and multiple intellectual disabilities, and comparatively low participation from people with intellectual disabilities in general, raises questions about whose perspectives shape research agendas on social care and related issues for minority ethnic communities (Frankena et al. 2015). It is also notable that, although equality, diversity and inclusion in research is a stated focus of UKRI and other funders (National Institute for Health and Care Research 2025), this review found few studies with clearly disaggregated findings on minority ethnic populations in research on social care and intellectual disabilities. Addressing these research gaps will require significant change at the level of funding and research planning.

Research that explores the concerns of diverse minority ethnic communities is vital, if services for people with intellectual disabilities are to be developed based on comprehensive insight into local communities and their needs, rather than on stereotypes and assumptions. Conversely, addressing gaps in research is essential to give service providers and commissioners the evidence they need to develop culturally competent social care services. We recommend that future academic research explores structural and intersectional inequalities in social care, and how these impact access to services for people from a range of minority communities, in response to concerns of community and policy organisations that “structural racism and discrimination are still endemic within our services” (Burke and Ong 2021), p. 3. Future research should also address populations missing from these studies, including people with profound and multiple intellectual disabilities, people who do not engage with services, and under‐researched minority communities including Black, Jewish and GRT communities, people of dual heritage and migrant communities. More participatory and coproduced research is also needed, to centre the perspectives of people with intellectual disabilities from minority ethnic communities, their families and wider communities.

Beyond research, the review has implications for social care policy and practice, as explored in the Discussion. Across the evidence base, there are recommendations that services should be easier for people with intellectual disabilities and their families to navigate, and adequately resourced to support those who engage with them. Studies in this review also point to a lack of support for family carers from minority ethnic communities, including some who do not have the extended family networks that service providers may assume they do. Much of the evidence makes the case that services should develop forms of cultural competence that are relational, person‐centred and anti‐racist, engaging with local communities and their organisations.

## Author Contributions

**Naomi L. Jacobs:** led the review; literature search and analysis; wrote first manuscript draft; manuscript review and editing. **Francesca Ribenfors:** conceptualisation; supported literature search, methodology and review; writing – review and editing. **Christopher Hatton:** conceptualisation; supported literature search, methodology and review; writing – review and editing. **Lucy Dunstan:** conceptualisation; supported methodology; writing – review and editing. **Anne‐Marie Glasby:** conceptualisation; supported methodology; writing – review and editing.

## Funding

The Minority Interest research project is funded by the NIHR Research for Social Care programme (Grant Reference Number NIHR206547). The views expressed are those of the authors and not necessarily those of the NIHR or the Department of Health and Social Care.

## Conflicts of Interest

The authors declare no conflicts of interest.

## Data Availability

Further data supporting the findings of this rapid review are available from the corresponding author upon reasonable request.

## Endnotes

^1^
Intersectionality is a theory that explores how multiple dimensions of identity (ethnicity, disability, gender, sexuality, religion and others) (Crenshaw 1989) interact to impact people's lives, including their experience of services. As Leeson and Dunstan observe (Leeson and Dunstan 2023), p. 10, disability is commonly treated as the dominant marker of identity for people with intellectual disabilities, both by services and in wider society, with a focus on disability to the exclusion of other aspects of people's identity.

^2^
“Family” refers here to family members of adults with intellectual disabilities, regardless of whether or not they hold a caring role. The research question and review focused on the experiences of people with intellectual disabilities and their families as recipients of social care, rather than on the experiences of carers. As this review found, research with carers on this topic, both family and professional, is more developed than research on the experiences of people with intellectual disabilities themselves.

^3^
“South Asian” is used here as a broad descriptive category, following the Minority Rights Group (Minority Rights Group 2021), referring to people of Indian, Pakistani, Bangladeshi, Sri Lankan, Nepali, Bhutanese and Maldivian heritage, and some communities of Middle Eastern heritage. Table 1 gives details of specific populations per study.

^4^
Many but not all of the recommendations in this section come from the grey literature, which had a policy and practitioner focus; recommendations generally arose from primary research.
